# On the Multiplication of Vegetable Cells by Division

**Published:** 1849-01

**Authors:** 


### RECORD OF MEDICAL SCIENCE.

### ANATOMY AND PHYSIOLOGY.

*On the Multiplication of Vegetable Cells by Division.* By Prof. Mitscherlich.—Some interesting observations on this subject occur in a memoir read by Professor Mitscherlich before the Royal Academy of Berlin, on the development and Composition of the Confervæ. They accord with the account of the process given by Mohl, and confirmed by Henfrey, as to the mode in which the multiplication of vegetable cells takes place by simple division. The process commences by a doubling-inwards of the "primordial utricle," or lining membrane of the cell, which detaches itself from the proper cell-wall, and exhibits an hour-glass constriction round its middle. The constriction continues to increase, until the original cell cavity is divided into two parts, the communication between which is entirely closed up. Between the two layers of the primordial utricle thus folded in, a new layer of cell-membrane is subsequently formed; and thus the two new cells are at last completely divided from one another. The opinions of observers are becoming more and more in favour of the view, that multiplication by the cell-division is the regular mode of increase in vegetating or growing parts. On the other hand it is also generally agreed that spores, pollen and embryos are produced by free cell-formation from nuclei.—*Brit. and For. Med. Chir. Rev. from Annals of Natural History, June,* 1848.

